# Role of Molecular Chaperones in Carcinogenesis: Mechanism, Diagnosis, and Treatment

**DOI:** 10.1155/2020/7437629

**Published:** 2020-04-20

**Authors:** Everly Conway de Macario, Alessandro Pitruzzella, Agata Grazia D'Amico

**Affiliations:** ^1^Department of Microbiology and Immunology, School of Medicine, University of Maryland at Baltimore-Institute of Marine and Environmental Technology (IMET), Columbus Center, Baltimore, MD 21202, USA; ^2^Euro-Mediterranean Institute of Science and Technology (IEMEST), Palermo 90139, Italy; ^3^Department of Biomedicine, Neuroscience and Advances Diagnosis (BIND), Section of Human Anatomy, University of Palermo, Palermo 90127, Italy; ^4^Consorzio Universitario Caltanissetta, Caltanissetta 93100, Italy; ^5^Department of Human Science and Promotion of Quality of Life, San Raffaele Open University of Rome, Rome, Italy

The role of molecular chaperones in carcinogenesis is a central theme of many current research efforts worldwide. It is pertinent to clarify that while various chaperones are Hsps, many are not, and vice versa not all Hsps are chaperones. An example of the latter is Hsp32, better known as heme oxygenase-1 (HO-1), which is dealt with in one of the articles in this Special Issue because of its probable involvement in certain types of carcinogenesis. Likewise, long noncoding RNAs are currently emerging as modulators of metastasization, and one contribution deals with this interesting new issue in Oncology.

While the involvement of chaperones in cancer progression has been extensively reported, the mechanism of their participation in carcinogenesis is largely unknown. Nevertheless, extant information is enough for considering these molecules as a promising biomarker for diagnosis and patient monitoring in some types of cancer and also promising to be used as therapeutic targets or agents. The aim of this Special Issue is to gather information about these themes.

Characterization of mechanisms underlying the role of molecular chaperones in cancer is an emerging issue in the oncology field. Molecular chaperones are involved in many biochemical pathways essential for tumor cell survival, and this makes them candidates for consideration as key players in the biochemistry of cancer. Therefore, the development of tools for diagnosis and treatment targeting chaperones is currently an active discipline within oncology.

Some malignant tumors can be classified as “chaperonopathies by mistake” because the molecular chaperones in the cancer cells contribute to their proliferation and mediate their resistance against antitumor defenses and facilitate metastasization. Chaperones are thus helping the “enemy” so to speak and are therefore “mistaken.” These chaperones work for the tumor rather than to defend the host. Efforts must be directed toward finding ways to eliminate or block these “mistaken” chaperones. This strategy of negative chaperonotherapy is currently being incorporated to the battery of other approaches such as chemo- and immunotherapy to treat cancer.

In this Special Issue, various examples of tumors in which Hsp-chaperones and a Hsp that is not a chaperone play a noteworthy role are discussed along with the potential of chaperonotherapy.

The review by Das et al. highlights recent advances and perspectives in Hsp-based cancer immunotherapy. The importance of research on the role of Hsp-chaperones, specifically Hsp27, Hsp60, Hsp70, and Hsp90, in modulating carcinogenesis is discussed, emphasizing their critical role in determining the balance between protective and destructive immunological responses within the tumor microenvironment; the possibilities involving Hsp27, Hsp70, and Hsp90 are clearly schematized in the first figure of the article, while pathways of presentation of tumor antigens by Hsps to antigen-presenting cells (APCs) are summarized in the second figure. Along this line of thought, Hsps are seen as an effective therapeutic option for some malignant tumors, including melanoma. Recent progress in this field is discussed, including anticancer vaccines, specifically those based on Hsp70 and Hsp90 are summarized in Table 1 of the article. These vaccines have been shown to be active against a spectrum of tumor antigens since they induce T-lymphocyte activation as well as stimulation of antigens uptake by APCs.

In the review by Fucarino et al., emphasis is given to the study of the molecular mechanisms in carcinogenesis that involve chaperones. Specifically, they focus on the chaperonin Hsp60 alone or in complex with Hsp10, and its implication in lung cancer, and also analyze the broad set of Hsp60 interactors, some of which are listed in Table 1 of the article. Noteworthy is the relationship of the fragile histidine triad (FHIT) protein, a tumor-suppressor factor, with Hsp60 and Hsp10 in complex, in mitochondria. FHIT, like many other mitochondrial proteins, depends for its correct folding on the Hsp60/Hsp10 chaperoning complex; it is, therefore, possible that a chaperonopathy affecting either one or the other of these two chaperonins will result in a decrease of tumor suppression, namely, will favor tumor growth. This is certainly a topic for future investigation because it may help find ways to apply positive chaperonotherapy to oppose tumor progression by administering Hsp60 and/or Hsp10 or boosting their activities, thereby reenforcing the chaperoning of FHIT protein correct folding, which will thus become an effective tumor suppressor. Continuous stress conditions in the respiratory mucosa, such as cigarette smoking, increase Hsp60 chaperonin levels outside mitochondria. High levels of this chaperonin are associated with tumor deterioration in some cancer types, but in other types, the reverse is seen, namely, tumor progression is favored probably due to inhibition of apoptosis and senescence in tumor cells by the chaperonin.

The paper by Wu et al. examines the effects of long noncoding RNA lnc-TLN2-4:1 in gastric cancer (GC). Forty-nine patients were recruited for this study who had not been submitted to chemotherapy before and were followed for four years. The authors demonstrate that lnc-TLN2-4:1 is decreased in GC tissue compared with matched normal tissue and is involved in poor overall survival rates of GC patients. Moreover, they show that lnc-TLN2-4:1 overexpression inhibits GC cell migration and invasion, but does not affect GC cell proliferation, suggesting its involvement as a tumor suppressor of GC metastases. In conclusion, Inc-TLN2-4:1 is a suppressor of metastasization and when it is under-regulated like in GC the survival rate of patients is poor.

The paper by Castruccio Castracani et al. presents data on the effect of estradiol E2 in human glioblastoma-multiforme (GBM) cells, focusing on proliferative capacity and mitochondrial functions. Expression of genes involved in mitochondrial biogenesis, oxidative phosphorylation, and dynamics was measured. Also, nuclear translocation of Nrf2 was assessed. The results showed that E2 increases the proliferation of glioblastoma cells and changes the expression of various genes among those investigated, e.g., those involved in oxidative phosphorylation. E2 also increased nuclear translocation of Nrf2 resulting in the induction of one of its target genes, hemeoxygenase-1, which is associated with the increase of both, chemoresistance and tumor-cell proliferation. These data show that E2 induces GBM proliferation and enhances its mitochondrial physiology, both of which certainly play a role in the well-known resistance of this tumor to all kinds of therapies, and open new avenues for developing novel treatment strategies.

In the paper by Kam et al., the authors report on the compound 2-methoxyestradiol as a potential anticancer agent, using as a model the A375 melanoma cells. They also studied the effect of polyphenol ferulic acid using the same cells. By applying MTT, flow cytometry, and Western blotting, the authors examined the molecular mechanisms underpinning the compounds' actions. These are partly related to the reduction of Hsp60 and Hsp90 levels and the induction of nitric oxide in A375 melanoma cells. The authors did not observe any changes in Hsp70 levels after 2-methoxyestradiol and ferulic acid treatment separately or in combination. This information is pertinent to evaluate chemoresistance mechanisms since Hsp70 accumulation reduces induction of cancer-cell death and decreases the antitumor efficacy of some therapeutic agents.

